# Assessment of DNA methylation profiling and copy number variation as indications of clonal relationship in ipsilateral and contralateral breast cancers to distinguish recurrent breast cancer from a second primary tumour

**DOI:** 10.1186/s12885-015-1676-0

**Published:** 2015-10-09

**Authors:** Katie T. Huang, Thomas Mikeska, Jason Li, Elena A. Takano, Ewan K A Millar, Peter H. Graham, Samantha E. Boyle, Ian G. Campbell, Terence P. Speed, Alexander Dobrovic, Stephen B. Fox

**Affiliations:** 1 Molecular Pathology Research and Development Laboratory, Department of Pathology, Peter MacCallum Cancer Centre, St. Andrew’s Place, East Melbourne, VIC 3002 Australia; 2 Department of Pathology and Sir Peter MacCallum Department of Oncology, University of Melbourne, Grattan Street, Parkville, VIC 3010 Australia; 3 Translational Genomics and Epigenomics Laboratory, Olivia Newton-John Cancer Research Institute, Studley Road, Heidelberg, VIC 3084 Australia; 4 Bioinformatics, Peter MacCallum Cancer Centre, St. Andrew’s Place, East Melbourne, VIC 3002 Australia; 5 South Eastern Area Laboratory Service (SEALS), St. George Hospital, Gary Street, Kogarah, NSW 2217 Australia; 6 The Kinghorn Cancer Centre & Garvan Institute of Medical Research, 384 Victoria Street, Darlinghurst, NSW 2010 Australia; 7 School of Medicine and Health Sciences, University of Western Sydney, Narellan Road, Campbelltown, NSW 2560 Australia; 8 Faculty of Medicine, University of NSW, High Street, Kensington, NSW 2052 Australia; 9 VBCRC Cancer Genetics Laboratory, Peter MacCallum Cancer Centre, St. Andrew’s Place, East Melbourne, VIC 3002 Australia; 10 Bioinformatics Division, Walter and Eliza Hall Institute of Medical Research, 1G Royal Parade, Parkville, VIC 3052 Australia; 11 School of Cancer Medicine, La Trobe University, Bundoora, VIC 3084 Australia

**Keywords:** DNA methylation, Comparative genomic hybridisation, Ipsilateral, Contralateral, Breast

## Abstract

**Background:**

Patients with breast cancer have an increased risk of developing subsequent breast cancers. It is important to distinguish whether these tumours are *de novo* or recurrences of the primary tumour in order to guide the appropriate therapy. Our aim was to investigate the use of DNA methylation profiling and array comparative genomic hybridization (aCGH) to determine whether the second tumour is clonally related to the first tumour.

**Methods:**

Methylation-sensitive high-resolution melting was used to screen promoter methylation in a panel of 13 genes reported as methylated in breast cancer (*RASSF1A*, *TWIST1*, *APC*, *WIF1*, *MGMT*, *MAL*, *CDH13*, *RARβ*, *BRCA1*, *CDH1*, *CDKN2A*, *TP73*, and *GSTP1*) in 29 tumour pairs (16 ipsilateral and 13 contralateral). Using the methylation profile of these genes, we employed a Bayesian and an empirical statistical approach to estimate clonal relationship. Copy number alterations were analysed using aCGH on the same set of tumour pairs.

**Results:**

There is a higher probability of the second tumour being recurrent in ipsilateral tumours compared with contralateral tumours (38 % versus 8 %; *p* <0.05) based on the methylation profile. Using previously reported recurrence rates as Bayesian prior probabilities, we classified 69 % of ipsilateral and 15 % of contralateral tumours as recurrent. The inferred clonal relationship results of the tumour pairs were generally concordant between methylation profiling and aCGH.

**Conclusion:**

Our results show that DNA methylation profiling as well as aCGH have potential as diagnostic tools in improving the clinical decisions to differentiate recurrences from a second *de novo* tumour.

**Electronic supplementary material:**

The online version of this article (doi:10.1186/s12885-015-1676-0) contains supplementary material, which is available to authorized users.

## Background

Patients with breast cancer are known to have a higher risk of developing a second breast tumour either in the affected (ipsilateral) or unaffected (contralateral) breast [1, 2]. When the second tumour is detected, it is important to determine whether the tumour is a *de novo* (new primary) tumour or a recurrence of the first tumour [3, 4] as the tumour staging and management for the patient will be different [5]. A recurrent breast tumour is known to be a predictor of developing breast metastasis and is associated with poor survival [6, 7], whereas a new primary may have a better outcome depending on the pathological features of the tumour [8].

Currently, histopathological features and clinical characteristics are most commonly used to determine the clonal origin of the tumours. These include histological type, degree of differentiation, presence of an *in situ* component, evidence for metastatic spread and the interval between tumour onsets [3]. However, tumours of distinct clonal origins may still have very similar histological features.

The use of molecular analysis can supply additional criteria to distinguish *de novo* second tumours from recurrent tumours. Goldstein *et al.* demonstrated that whereas six out of eight ipsilateral sample pairs (75 %) were clonally different using a loss of heterozygosity (LOH) assay, the morphology of the tumour pairs was similar [9]. They also found that approximately 42 % of sample pairs had discrepancies between histopathological classification and molecular classification using LOH patterns. Thus, using histopathological features and clinical characteristics alone may not correctly identify the relationship between *de novo* and recurrent breast cancer [9]. Recently, several other molecular methodologies have been assessed for their usefulness in determining the clonal relationship of the tumours. For example, microsatellite instability patterns [10–13], the pattern of X chromosome inactivation [14], and *TP53* mutations [15] have been used. However, the best differentiation between *de novo* and recurrent tumours to date has been given by allelic imbalance profiles that result from LOH and tumour heteroploidy as measured by aCGH [10, 12, 13, 16].

DNA methylation changes are widespread in cancer and as methylation patterns are often clonally inherited [17], they can also be used to determine the clonal relationship of tumours. It is expected that tumours of the same clonal origin will have closely related DNA methylation patterns and profiles. In this study, we set out to compare DNA methylation profiling and aCGH as tools to distinguish between *de novo* and recurrent tumours.

## Methods

### Sample collection and DNA preparation

Formalin-fixed, paraffin-embedded (FFPE) samples for 16 pairs of ipsilateral and 13 pairs of contralateral breast cancers diagnosed from 1997 to 2007 were obtained from the St. George Hospital, Sydney, Australia. Ethics approval was granted by St. George Hospital Human Research Ethics Committee (07/60) with a waiver of informed consent to obtain archival samples. In addition our study complies with the current laws of Australia and ethics approval was obtained from the Peter MacCallum Cancer Centre Ethics Committee (03/90). Each pair included the primary tumour and the second tumour. The de-identified haematoxylin-eosin stained sections were reviewed by a pathologist and representative tumour areas were marked and needle-macrodissected and genomic DNA was extracted by 3 days incubation at 56 °C in buffer ATL (Qiagen, Hilden, Germany) with 20 mg/μL proteinase K (Qiagen) added daily, followed by QIAamp DNA Blood Mini Kit (Qiagen) spin columns according to the manufacturer’s instructions.

### Bisulfite modification

Genomic DNA (500 ng) was bisulfite modified using the MethylEasy™ Xceed (Human Genetic Signatures, North Ryde, Australia) according to the manufacturer’s instructions. The modified DNA was eluted twice in 25 μL of EB buffer. CpGenome™ Universal Methylated DNA (Chemicon/Millipore, Billerica, MA) and peripheral blood mononuclear DNA were used as the methylated and unmethylated controls, respectively. DNA methylation standards (5, 10, 25 and 50 %) made by diluting the fully methylated control in the unmethylated DNA were used as controls. Whole-genome amplification (WGA) was also used to make a fully unmethylated control and performed as described previously [18].

### Methylation-sensitive high-resolution melting (MS-HRM)

MS-HRM was used to detect methylation in bisulfite modified samples according to the sequence-dependent thermostability in which the level and presence of heterogeneous methylation can be detected [19, 20]. A panel of 13 genes that have been reported to be methylated in breast cancer (*RASSF1A*, *TWIST1*, *CDH13*, *APC*, *MAL*, *GSTP1*, *WIF1*, *RARβ*, *BRCA1*, *CDKN2A*, *TP73*, *CDH1* and *MGMT*) was chosen for screening the breast carcinoma samples. *MLH1*, which is not methylated in breast cancer, was included as a negative control for methylation. MS-HRM primers were used as previously described [21, 22] except for *CDH13* and *GSTP1*. All MS-HRM assays were designed to amplify amplicon sizes around 100 bp to enable amplification from the majority of FFPE samples. Primers were designed according to the principles described previously [23]. The *CDH13* MS-HRM primers were 5’-TxTGGTTTTTACGGAAAATATGTTTAGTGTA-3’ (forward) and 5’-AATTCTCGACTACATTTTATCCGACTAAAA-3’ (reverse). The 93 bp amplicon (GenBank AC092351 nucleotides 82660620–712) contains six CpGs. The *GSTP1* MS-HRM primers were 5’-GGGGCGGGATTATTTTTATAAGGTT-3’ (forward) and 5’-GAATTAACCCCATACTAAAAACTCTAAACC-3’ (reverse). The 140 bp amplicon (GenBank AP001184, nucleotides 67351239–337) contains 14 CpGs.

PCR amplification and high-resolution melting analysis were performed on the Rotor-Gene Q (Qiagen). PCR conditions for each gene are described in Table 1. PCR was performed using a final volume of 20 μL. The reaction mixture consisted of 1 × PCR buffer (Qiagen), 2.5–4.0 mmol/L of MgCl_2_, 200 μmol/L of each dNTP, forward and reverse primers, 5 μmol/L of SYTO9 intercalating dye (Invitrogen, Carlsbad, CA), 0.5U of HotStarTaq DNA polymerase (Qiagen) and 10 ng of bisulfite modified DNA. HRM was performed directly after PCR amplification. HRM consisted of an inactivation step at 97 °C for 1 min, rapid cooling to 72 °C (or 69 °C), then melting of the sample from 72 °C to 95 °C with temperature rising by 0.2 °C per second and holding for 1 s after each stepwise increment for all assays. In each assay, fully methylated, WGA DNA (unmethylated), different DNA methylation percentage dilution standards and no template controls were also performed. All assays were performed in duplicate.

**Table 1:** PCR amplification conditions for the MS-HRM assays

Gene	MgCl_2_ concentration (mmolL^−1^)	Primer concentration (nmolL^−1^)	Cycling time (sec)	Annealing temperature (°C)
*APC*	2.5	F - 200	10, 15, 20	58.5
		R - 300		
*BRCA1*	4.0	F - 250	10, 10, 20	61.0
		R - 250		
*CDH1*	2.5	F - 200	10, 10, 20	63.5
		R - 200		
*CDH13*	3.0	F - 300	10, 20, 25	58.0
		R - 400		
*CDKN2A* (*P16*)	2.5	F - 300	10, 10, 20	70.0
		R - 200		
*GSTP1*	2.5	F - 200	10, 10, 20	64.5
		R - 200		
*MAL*	2.5	F - 200	10, 10, 20	59.0
		R - 200		
*MGMT*	4.0	F - 250	10, 20, 20	60.0
		R - 250		
*MLH1*	3.5	F - 200	10, 15, 20	59.0
		R - 300		
*TP73*	3.0	F - 300	10, 15, 20	55.0
		R - 200		
*RARβ*	3.0	F - 200	10, 10, 20	66.0
		R - 300		
*RASSF1A*	3.0	F - 300	15, 25, 20	65.0
		R - 200		
*TWIST1*	2.5	F - 200	10, 10, 20	52.0
		R - 300		
*WIF1*	3.0	F - 400	10, 20, 25	53.0
		R - 400		

### Methylation scoring

Methylation for each gene was considered positive when it was above 10 %. Setting a cut off point is important for methylation scoring as low-level endogenous methylation may also be found in normal breast tissue. This was observed in some patient matched normal breast tissues, where low levels of methylation (less than 10 %) can be detected for some genes in these matched normal breast tissues (KTH, TM, AD unpublished results). Samples giving non-reproducible melting profiles and late PCR amplification were scored as uninformative. Methylation was independently scored by KTH and TM, with a third opinion from AD where scoring was discrepant.

### Analysis of DNA methylation data

The assumption was made that due to the clonal inheritance of methylation, clonally related tumours should have methylation patterns that closely resemble each other. Previous reports have shown that methylation levels increase during tumour progression in breast cancer (i.e., *in situ* carcinoma to invasive carcinoma) [24]. Therefore, the further assumption was made that an unmethylated marker in a primary tumour has a higher probability to be methylated in a clonally related second tumour than a methylated marker becoming unmethylated.

*Log odds ratios* were calculated for each pair of tumours as a measurement of likelihood of recurrence using methylation data. In the following formulas, *R* stands for recurrence, i.e., the two given tumours come from the same origin; $\overline{R}$ for non-recurrence, i.e., different origins; *M* represents methylated; and $\overline{M}$ represents unmethylated. *p*_*i*_ represents the probability of gene *i* being methylated, where the values of *p*_*i*_ or each of the 13 genes have been estimated from the literature (see below). Thus, using *p*_*i*_, the probabilities of a gene being A) methylated in both tumours, B) unmethylated in both tumours and C) methylated in only one of the tumours can be calculated, conditional on recurrence/non-recurrence.

Given non-recurrence, these probabilities are:

**Table:** 

**Non-recurrence**	$\overline{M}$ (secondary)	*M* (secondary)
$\overline{M}$ (primary)	(1 − *p*_*i*_)^2^	*p*_*i*_(1 − *p*_*i*_)
*M* (primary)	*p*_*i*_(1 − *p*_*i*_)	*p*_*i*_^2^

Given recurrence, the probability of losing methylation over the course of progression from primary to secondary tumour, *γ* has been estimated from the aCGH data to be 0.05. On the other hand, the probability of gaining methylation, which is expected to be higher as stated in our assumption above, is estimated to be 2 *γ*

**Table:** 

**Recurrence**	$\overline{M}$ (secondary)	*M* (secondary)
$\overline{M}$ (primary)	(1 − 2*γ*)(1 − *p*_*i*_)	2*γ*(1 − *p*_*i*_)
*M* (primary)	*γp*_*i*_	(1 − *γ*)*p*_*i*_

These tables are used to calculate the probabilities of observing the methylation status of each gene as seen in the methylation data. For example, if primary tumour A is unmethylated and secondary tumour B is methylated in gene *i*, then, according to the recurrence and non-recurrence probability tables respectively,

Pr(*x*_*i*_|*R*) = 2*γ*(1 − *p*_*i*_) and $$ \Pr \left({x}_i\Big|\overline{R}\right)={p}_i\left(1-{p}_i\right) $$

where *x*_*i*_ represents the observed methylation status of gene *i* in the MS-HRM data.

The Log-Odds Ratios (LR) *are then* calculated by the formula described below.

$$ LR={\displaystyle \sum_{i=1}^{13}}lo{g}_e\frac{ \Pr \left({x}_i\Big|R\right)}{ \Pr \left({x}_i\Big|\overline{R}\right)} $$

Two methods were then used to assess the LR values, and subsequently the likelihood of recurrence: an empirical approach and a Bayesian approach. The empirical approach unbiasedly utilises the methylation data to statistically assess clonal relationship by generating a null distribution of log-ratios representing the non-recurrence population, without making pre-assumptions about the likelihood of recurrence. However, since the strength of this approach is limited by the small sample size and the limited number of gene markers, more accurate classifications of *de novo* versus recurrence can be achieved by incorporating prior knowledge, hence the Bayesian approach.

### Method 1: Bayesian inference

The key information required to calculate the likelihood is the probability of each of the 13 genes being methylated in tumours. The average methylation frequency of each gene in breast cancer was obtained from the literature [24–41]. The published results were reviewed and selected by two individuals together (KTH and TM) for the final decision (Additional file 1: Table S1). The aCGH data generated in the study was also used in the calculating the log odds ratios.

When the chance between recurrence and non-recurrence is not 50/50, then Bayesian inference was used to calculate posterior LR, or PLR, based on the prior knowledge of the chance of recurrence/non-recurrence. Applying Bayes’ theorem of conditional probability on the above LR formula, we get

$$ PLR=lo{g}_e\frac{ \Pr (R)}{ \Pr \left(\overline{R}\right)}+LR $$

The prior probability of recurrence, i.e., Pr(*R*) were set at 0.75 for ipsilateral samples and 0.145 for contralateral samples. These values were obtained from literature that used molecular assays of aCGH, LOH and p53 mutations to differentiate recurrent and *de novo* tumours between the pair tumours [9, 12, 15, 16]. The frequency of recurrent tumours found in ipsilateral tumour pairs was about 75 % (ranged from 69–76 %), while the frequency of recurrent tumour found in contralateral tumour pairs was about 14.5 % (averaged value of 14 % and 15 %).

A sample can then be called recurrence or *de novo* depending on the PLR value: *PLR* > 0 suggests recurrence, while *PLR* < 0 suggests non-recurrence. These two inequalities are equivalent to $LR>-lo{g}_e\frac{ \Pr (R)}{ \Pr \left(\overline{R}\right)}$ and $LR<-lo{g}_e\frac{ \Pr (R)}{ \Pr \left(\overline{R}\right)}$ respectively. Substituting in the probabilities of recurrences for ipsilateral samples gives *LR* > − 1.099 and *LR* < − 1.099. For contralateral samples, these are *LR* > 1.774 and *LR* < 1.774 respectively.

### Method 2: An empirical approach

The methylation data was also used to obtain an empirical (i.e., prior knowledge was not used) null distribution of log ratios representing the non-recurrence population. Cross comparison between tumours from the 29 individuals gave us 3248 (pairing up each tumour with those from other individuals gives 2 × 29 × 2 × 28 = 3248 combinations) pairwise comparisons of non-recurrent cases. The LRs obtained from these comparisons form a null, or background, distribution from which *P*-values were calculated. The plot follows an approximately normally distribution (Additional file 2: Figure S1). *P*-values were calculated by counting the number of cases in the null with LRs larger than the LR of interest, and then dividing that number by the total number of null cases. The *P*-value cut-off for indicating the significance of recurrence was set at 0.05 in the study (i.e., a *P*-value <0.05 indicates the second tumour is likely to be clonally related to the first tumour).

### Array comparative genomic hybridization (aCGH) data generation and analyses

Genomic DNA (500 ng) from the same batch of tumour DNA as used in methylation profiling was analysed using the Agilent oligonucleotide array-bases CGH (4x microarray) following the manufacturer’s instructions (Agilent Technologies, Santa Clara, CA). In brief, genomic DNA of samples and female reference DNA (a normal control DNA) (Promega, Madison, WI) were first fragmented for 30 s at 95 °C and 30 min at 95 °C respectively, and then the reference and sample DNA were labeled with ULS-Cy3 and ULS-Cy5 dye with the ratio 1 μL per 1 μg DNA, respectively. The non-reacted Cy-ULS dyes were removed using Agilent KREApure columns to reduce possible background noise for array screening. Optimal Cy5 degree of labeling (range between 0.75 % and 2.5 %) with a Cy3 minus Cy5 range between 1 % and 2 % were used as a quality control guideline for sample labeling before hybridizing to the array. Samples and references DNA were hybridized on to the microarray at 65 °C for 40 h, and then washed and scanned. aCGH result analyses was performed using the Partek® Genomics Suite™ version 6.03 (Partek Inc., St. Louis, MO).

### Statistical analysis

Statistical analyses were performed using GraphPad Prism version 5.01 (GraphPad Software, San Diego, CA). Comparisons of age and tumour size between ipsilateral and contralateral groups were evaluated using the unpaired Student’s *t*-test and nonparametric Mann–Whitney *U* test, respectively. Fisher’s exact test was used to examine the association between ipsilateral and contralateral groups with recurrent and *de novo* clonality. A two-tailed *p*-value of <0.05 was considered to be significant for each comparison.

## Results

### Patient characteristics

The clinical and pathological features of the patients in this study are summarised in Table 2. A trend was observed for patients with ipsilateral tumours to develop their primary tumours at an earlier median age than patients with contralateral tumours (54 vs. 59 years), although it was not significant at the 5 % level (*P* = 0.08). The median age of onset for developing a second tumour was also earlier for ipsilateral patients compared with contralateral patients (59 vs. 68 years) (*P* = 0.07). However, the median time interval for the second tumours to develop was similar between ipsilateral patients (4 years; range, 0–14 years) and contralateral patients (5 years; range, 1–9 years) (*P* = 0.60) after the initial breast tumour diagnosed.

**Table 2:** Clinicopathological features of the ipsilateral and contralateral breast cancers

	Ipsilateral (*n* = 16)	Contralateral (*n* = 13)
Age of onset (years)		
Primary		
Median	54	59
Range	37 – 75	43 – 74
*P*-value		0.082
Second tumour		
Median	59	68
Range	40 – 82	45 – 80
*P*-value		0.070
Age interval (years)^a^		
Median	4	5
Range	0 – 14	1 – 9
*P*-value		0.604
Tumour size		
Primary		
Median (mm)	17.8	17
Range (mm)	6 – 50	10 – 40
*P*-value		0.711
Second tumour		
Median (mm)	13.5	–
Range (mm)	0.3 – 75	–

^a^Time interval between first and second tumour onset

### DNA methylation patterns in ipsilateral and contralateral sample pairs

Methylation of 13 cancer related genes was assessed in 16 ipsilateral and 13 contralateral breast cancer pairs using MS-HRM (Table 3). In addition, promoter methylation of *MLH1* was assessed and treated as a negative methylation control, as it is known to be unmethylated in breast carcinomas.

**Table 3:**
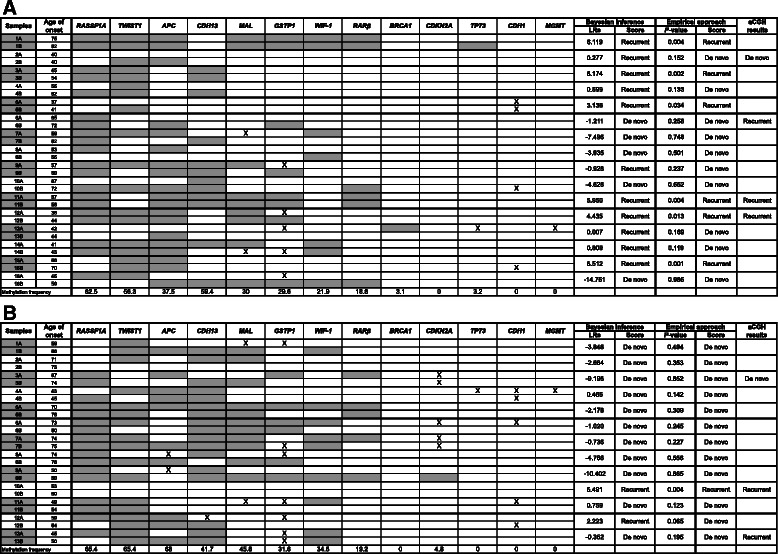
DNA methylation profile by MS-HRM analysis in (A) 16 ipsilateral and (B) 13 contralateral breast carcinomas

The methylation frequency of each gene and the age onset of each sample are included in the table. Results of tumour origin of the paired tumours that scored using MS-HRM and aCGH were stated on the left side of the table. Grey represents methylation and X represents samples that did not amplify

**Fig. 1:**
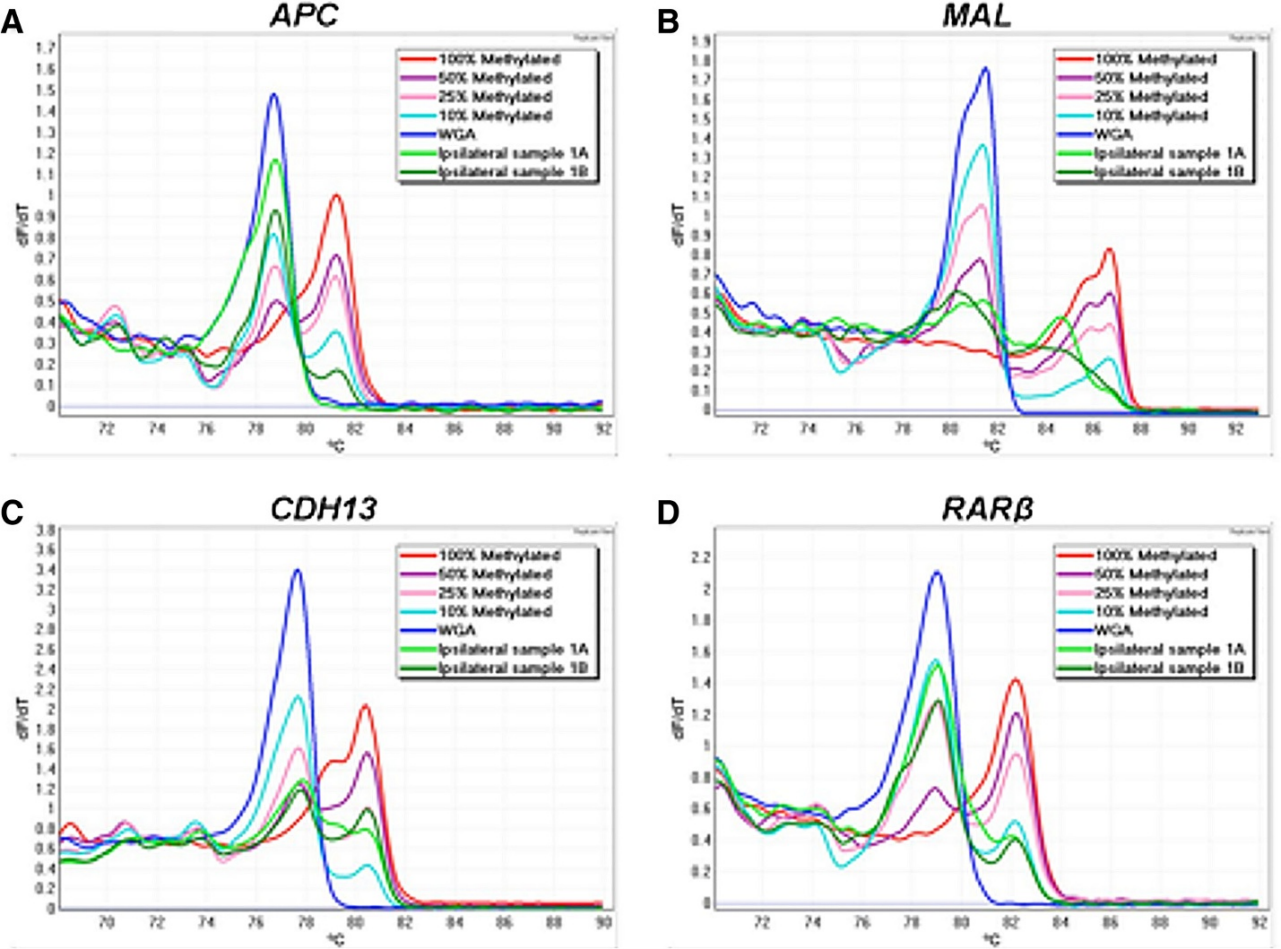
Examples of MS-HRM analysis of **a** *APC*, **b** *MAL*, **c** *CDH13* and **d** *RARβ* genes in ipsilateral sample 1. The figure shows negative first derivative (*T*_*m*_) melting curves of the MS-HRM profiles. MS-HRM differentiates the methylated DNA from the unmethylated DNA based on the sequence-dependent thermostability. Fully methylated samples melt later than the unmethylated WGA samples as there are cytosines retained in the sequence after bisulfite modification. Standards with different methylation levels (10 %, 25 % and 50 %) were prepared by mixing the fully methylated DNA with fully unmethylated DNA. Ipsilateral sample 1A represents the first tumour and 1B represents the second tumour

Eleven cancer related genes were found to have methylation (defined as more than 10 % methylation) in at least some of the tumours: *RASSF1A* (64 %), *TWIST1* (61 %), *CDH13* (51 %), *APC* (50 %), *MAL* (35 %), *GSTP1* (30 %), *WIF1* (26 %), *RARβ* (19 %), *BRCA1* (2 %), *CDKN2A* (2 %) and *TP73* (2 %). Two genes *CDH1* and *MGMT* were scored as unmethylated for all the samples in this study as either no methylation or very low-level methylation was detected. No promoter methylation of *MLH1* was found in any of the samples. Examples of the MS-HRM results are shown in Fig. 1.

### Determining clonal relationships using DNA methylation profiling

DNA methylation patterns of the paired tumours were compared to assess the likelihood that the second tumour had arisen from the first tumour. Some tumour pairs, in particular ipsilateral pairs, showed very similar DNA methylation patterns between the first and the second tumours, whereas others showed markedly different methylation patterns between the paired tumours. For example, ipsilateral tumour pair 1 showed highly similar methylation patterns, where all the genes that were methylated in the primary tumour were also methylated in the second tumour with additional methylation in *TP73*. On the other hand, contralateral pair 2 showed methylation of *CDH13*, *MAL* and *TWIST1* in the first tumour but no methylation of any of the marker panel was found in the second tumour.

To objectively score whether both tumours are clonally related in origin using DNA methylation patterns between the paired tumours, log odds ratios were calculated as a measurement of likelihood of recurrence. An empirical approach was employed to assess the methylation data against an estimated null distribution without making a prior assumption on the likelihood of recurrence. Eight percent of contralateral tumours and 38 % of ipsilateral tumours were called recurrent with a significance of *P* <0.05 using this approach. The much higher proportion of ipsilateral cases with recurrence is consistent with expectation, supporting the use of methylation data as a tool for assessing clonal relationship.

We also applied Bayesian inference to determine the clonal relationship between each pair of tumours based on their methylation patterns. Posterior log-odds ratios, indicating either positive or negative clonal relationships, were calculated for each tumour pair using previously obtained methylation frequency data for each of the genes (Additional file 1: Table S1). The prior probabilities of a tumour being recurrent for contralateral and ipsilateral pairs were estimated to be 0.145 and 0.75 respectively.

Using Bayesian inference, it was determined that there were eleven recurrent pairs (69 %) and five *de novo* pairs (31 %) in ipsilateral tumours, compared with two recurrent pairs (15 %) and eleven *de novo* pairs (85 %) in contralateral tumours (Table 4a). Using the empirical approach, it was determined that there were six recurrent pairs (38 %) and ten *de novo* pairs (62 %) in ipsilateral tumours, compared with one recurrent pair (8 %) and twelve *de novo* pairs (92 %) in contralateral tumours (Table 4b). Although not all results analysed by both approaches were concordant (79.3 %), in both cases the results are consistent with the expectation that ipsilateral tumours had a higher chance of being recurrent and contralateral tumours had a higher chance of being *de novo*.

**Table 4:** Summary of deduced clonal origins of ipsilateral and contralateral tumour pairs using (a) a Bayesian inference approach and (b) an empirical approach

	Recurrent	*De novo*	Total
a			
Ipsilateral	11 (69 %)	5 (31 %)	16 (100 %)
Contralateral	2 (15 %)	11 (85 %)	13 (100 %)
b			
Ipsilateral	6 (38 %)	10 (62 %)	16 (100 %)
Contralateral	1 (8 %)	12 (92 %)	13 (100 %)

### Comparison of genomic copy number in ipsilateral and contralateral tumour pairs

Informative results using array comparative genomic hybridization (aCGH) were obtained from 4 ipsilateral pairs and 3 contralateral pairs whereas the DNA quality from the remaining 17 sample pairs was too poor to achieve adequate labeling for array screening after at least two attempts. In general, the aCGH data from the second tumour was better quality with less noise than the data from the primary tumour consistent with the better quality of the DNA from the younger FFPE tumour block.

The copy number was compared between each tumour pair to determine the possible clonal relationship. Of the seven sample pairs, two of the second tumours were determined as *de novo* (one from ipsilateral pairs and one from contralateral pairs) and five of the second tumours were determined as recurrent (three from ipsilateral pairs and two from contralateral pairs). Array CGH results are summarised in Table 5.

**Table 5:** Gene copy number variation results from aCGH on seven sample pairs

Sample		Loss of chromosome	Gain of chromosome	Recurrent/*De novo*	Note
Ipsilateral 2	First tumour			Likely to be *de novo*	
	Second tumour	2p, 3p (partial),	7q, 8q, 10p (partial), 19p		
Ipsilateral 6	First tumour	3p (partial)	17q (partial)	Likely to be recurrent	The second tumour still retains the variation of the primary tumour, especially gain of 17q12 (Her2)
	Second tumour	3p (partial), 4q, 8p, 9p, 10p, 11q, 13, 18	1q (partial), 12p, 14p, 17q (partial), 19p		
Ipsilateral 11	First tumour		5q (partial), 8q (partial), 17q (partial)	Recurrent	Whole genome variation of primary and second tumour overlaid exactly
	Second tumour		5q (partial), 8q (partial), 17q (partial)		
Ipsilateral 12	First tumour	6q, 11q, 12q, 13	1q, 11p (partial), 12p, 12q (partial), 19	Likely to be recurrent	Similar overall patterns in chromosome 11, 12 and 19
	Second tumour	2q (partial), 3p (partial), 4, 6q, 7q (partial), 11q, 12q, 13	1q, 11p (partial), 12p, 12q (partial), 15, 19		
Contralateral 3	First tumour	6q, 11q, 18	1q, 11p, 17q (partial)	Likely to be *de novo*	
	Second tumour	6q, 16q, 22	1q, 16p, Xq		
Contralateral 10	First tumour	3p	6p, 8q	Likely to be recurrent	Similar overall patterns, especially in chromosome 8, 21 and 22
	Second tumour		6p, 8q		
Contralateral 13	First tumour		3p (partial), 8q, 11q (partial)	Likely to be recurrent	
	Second tumour		3p (partial), 8q, 11q (partial)		

**Fig. 2:**
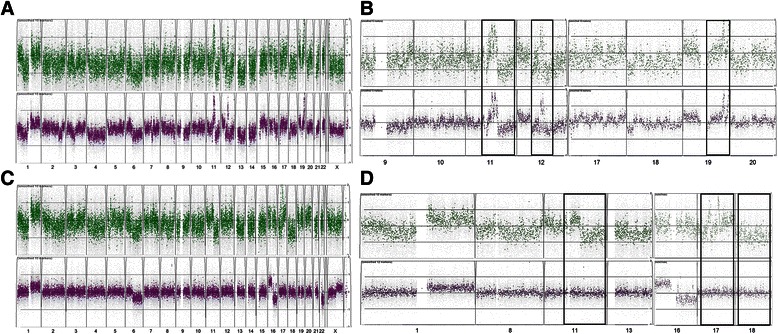
Examples of genomic aCGH profiles of a recurrent ipsilateral pair 12 with **a** whole genome profile and **b** individual chromosomes of 9, 10, 11, 12, 17, 18, 19 and 20; and a *de novo* contralateral pair 3 with **c** whole genome profile and **d** individual chromosomes of 1, 8, 11, 13, 16, 17 and 18. Primary tumour (green) on top and the second tumour (purple) on bottom. Most informative areas were highlighted by the solid box

Examples of aCGH results (ipsilateral pair 12 and contralateral pair 3) are shown in Fig. 2. In ipsilateral pair 12, the gains of chromosome 1q, part of 11q (including the cyclinD1 locus on11q13), 12p, part of 12q and 19 and losses of chromosome 6q, part of 11q, 12q and 13 were found in the primary tumour (Fig. 2a, b). These gains and losses were also found in the second tumour with the similar pattern or at the same position. Hence, the second tumour is highly likely to be recurrent from the primary. In contralateral pair 3, there were several gains and losses found in the primary tumour that was not found in the second tumour, such as gain of chromosome 11p and part of 17q and loss of chromosome 18. It is less likely for tumours of the same clonal origin to have different genomic copy number. Thus, the second tumour is likely to be a *de novo* tumour (Fig. 2c, d).

### Comparison between methylation profile and CGH microarray

The best differentiation between *de novo* and recurrent tumours up to now has been given by allelic imbalance profiles. Therefore, the clonal origin results determined by aCGH was used to reflect the actual tumour origin of the tumour pairs in order to calculate the predictive values of sensitivity, specificity, positive predictive value and negative predictive value for methylation results scored by both algorithms (Table 6).

**Table 6:** Predictive values of using methylation profiling with different algorithms to distinguish tumour origins (*n* = 7)

		Methylation		
		Recurrent	*De novo*	
aCGH	Recurrent	3	2	Sn = 0.60 (3/5)
	*De novo*	1	1	Sp = 0.50 (1/2)
		PPV = 0.75 (3/4)	NPV = 0.33 (1/3)	

Results were scored using Bayesian inference approach

*NPV* negative predictive value, *PPV* positive predictive value, *Sn* sensitivity, *Sp* specificity

We compared the clonal results of the tumour pairs determined using methylation profiling and aCGH data. A discrepancy was seen in three out of seven tumour pairs when comparing aCGH prediction to the methylation profiling prediction using the Bayesian inference formula.

## Discussion

Several types of molecular analyses have been previously used to assess the clonal relationships between ipsilateral and contralateral breast cancers. These include assays for genomic imbalance (by aCGH) and *TP53* mutation screening [3, 10, 15, 16]. We performed DNA methylation profiling of a set of genes commonly methylated in breast cancer to assess if this is of use to determine whether the first and the second tumour are clonally related.

It has been shown that individual breast cancers have a distinct profile of methylated genes [42]. Thus, we hypothesized that DNA methylation profiling either by itself or combined with genetic alterations using aCGH therefore might be a useful tool to distinguish between *de novo* and recurrent tumours.

The DNA methylation frequency of genes studied in our tumour cohort was compared with what has been reported in the literature for breast cancers (Additional file 1: Table S1). For example, *RASSF1A, CDH13 and RARβ* have been reported to be frequently methylated in breast cancer (average frequency of 71 %, 49 % and 21 % respectively, Additional file 1: Table S1), which is similar to our results of 64 %, 51 % and 19 % methylation in both ipsilateral and contralateral pairs respectively. However discrepant values such as *TWIST1* 61 % vs 26 %, *WIF1* 28 % vs 65 % and *CDH1* 0 % vs 28 % most likely represent variation in the region being examined and methodology. Interestingly, there was only one sample that was methylated for *BRCA1* (2 %), a frequency about ten times less than what is typically identified in the literature [26, 43] that is likely due to bias from a relatively small series.

Although *CDH1* has been reported to be frequently methylated in breast tumours and suggested as a biomarker for breast cancer, we did not identify any methylation of *CDH1*. Different methodologies have been used to detect the methylation status of *CDH1* in breast cancers and a wide range of methylation frequencies in breast cancers has been reported (range from 1 to 23 %). However, our result was consistent with both our previous MS-HRM data (1 %: unpublished) and Feng *et al.* who employed bisulfite pyrosequencing [31] and reported methylation at low levels in both malignant and normal breast tissue.

The methods we have used eliminate the biases intrinsic to traditional ways of determining tumour origins, which often involve subjective input from a pathologist (operator) and which have shown to be inaccurate. However, the accuracy of our methods is dependent on a prior knowledge on methylation frequencies (in the case of Bayesian approach) or the number of samples from which a null distribution is estimated (empirical approach). Nevertheless, while both methods had similar NPV, the empirical approach showed a better representation and PPV suggesting is the better predictor for tumor relatedness based on methylation profiling. However it must be noted that this conclusion is based on a small number of tumors and gene markers.

Nonetheless, this study has demonstrated by methylation profiling, that a proportion of subsequent breast tumours share similar methylation profiles with the first tumour indicating that these tumours are likely to be clonally related. As also anticipated, ipsilateral tumours have a higher probability of being recurrent compared with contralateral tumours, which had a higher chance of being *de novo* tumours. These results are consistent with reports in the literature using different methodologies that ipsilateral breast cancers mostly arise from a single breast cancer whereas contralateral breast cancers frequently represent different primary breast cancers [3, 15, 16]. Based on previous studies, approximately 75 % of second tumours are clonally related to the initial carcinoma in ipsilateral cases [9, 12, 16], compared to only ~15 % of contralateral [15, 16]. The frequency of recurrent and *de novo* tumours found in ipsilateral and contralateral cases reported in the literature are very close to the methylation results scored using the Bayesian inference approach in this paper. However, the recurrence rates called significant by our approach are lower than expected from the literature (15 % and 75 %).

Array CGH was used to validate the clonal predictions based on methylation but only a small number of samples were assessable by aCGH due to poor DNA quality. Nevertheless, informative aCGH results were obtained for seven sample pairs. In general, the later onset tumours had frequent additional genomic copy number alterations consistent with continued clonal evolution. Frequently altered genes in breast cancer can be useful as clinical markers, such as *HER2* or *CCND1*, which allows the identification of the clonal relationship in a tumour pair. For example, gain of *HER2* (17q12) was found in both primary and second tumours of ipsilateral pair 6, and gain of *CCND1* (11q13) was found in both primary and second tumours of ipsilateral 12.

Genome-wide DNA copy number variation in tumours has been widely studied and used as a reliable tool to differentiate between tumours. However, the applicability of the technique to FFPE DNA is limited by DNA fragmentation, which can preclude generation of interpretable data. It is for this reason that we developed assays using MS-HRM that can be designed to meet the challenges associated with analysis of poor quality FFPE samples.

## Conclusions

In summary, DNA methylation profiling using methodology compatible with degraded DNA has potential to be used as a diagnostic tool in improving the clinical decisions to differentiate recurrences from a second *de novo* tumour in FFPE samples.

## Additional files

Additional file 1: Table S1. Comparison of DNA methylation frequency of genes screened in breast carcinomas with the methylation frequency in literature. (DOCX 100 kb)
Additional file 2: Figure S1. Normally distributed test statistics generated using empirical null distribution of LRs. *N* = 3248 and bandwidth = 0.8926. (TIFF 115 kb)

## Abbreviations

aCGH: array comparative genomic hybridization
LOH: Loss of heterozygosity
FFPE: Formalin-fixed paraffin-embedded
WGA: Whole-genome amplification
MS-HRM: Methylation-sensitive high-resolution melting
PPV: Positive predictive value
NPV: Negative predictive value
