# Supplementary material for: Assessment of DNA methylation profiling and copy number variation as indications of clonal relationship in ipsilateral and contralateral breast cancers to distinguish recurrent breast cancer from a second primary tumour
Source: BMC Cancer. 2015 Oct 9;15:669. doi: 10.1186/s12885-015-1676-0 (PMC4600279; doi:10.1186/s12885-015-1676-0)
Supplement: Additional file 1: Table S1. — Comparison of DNA methylation frequency of genes screened in breast carcinomas with the methylation frequency in literature. (DOCX 100 kb) [file 12885_2015_1676_MOESM1_ESM.docx]

**Table S1. Comparison of DNA methylation frequency of genes screened in breast carcinomas with the methylation frequency in literature**

| **Gene** | **MS-HRM** | **Literature**  **(Average)** | **Methods used in literature** |
| --- | --- | --- | --- |
| ***RASSF1A*** | 64% | 71% | Pyrosequencing (Feng et al., 2007;  Pasquali et al., 2007), MethyLight (Cho et al., 2010); MSP (Sharma et al.,  2009); MS-HRM (unpublished data) |
| ***TWIST1*** | 61% | 26% | Quantitative multiplex MSP (Gort et  al., 2008); MethyLight (Cho et al.,  2010) |
| ***APC*** | 53% | 37% | Bisulfite sequencing/MSP (Jin et al.,  2001); MethyLight (Cho et al., 2010); MSP (Prasad et al., 2008) |
| ***CDH13*** | 51% | 49% | Pyrosequencing (Feng et al., 2007);  MSP (Toyooka et al., 2001); MS-HRM (unpublished data) |
| ***MAL*** | 38% | 66% | Bisulfite sequencing (Horne et al.,  2009); MS-HRM (unpublished data) |
| ***GSTP1*** | 31% | 47% | Pyrosequencing (Pasquali et al., 2007);  MSP (Esteller et al., 2001; Sharma et al., 2009); MALDI (Ronneberg et al.,  2008) |
| ***WIF1*** | 28% | 65% | Bisulfite sequencing/MSP (Ai et al.,  2006); MSP (Veeck et al., 2009) |
| ***RARβ*** | 19% | 21% | Pyrosequencing (Feng et al., 2007);  MethyLight (Cho et al., 2010); MSP (Sharma et al., 2009) |
| ***BRCA1*** | 2% | 20% | Southern blot (Bianco et al., 2000;  Dobrovic & Simpfendorfer, 1997); MethyLight (Cho et al., 2010); MSP (Esteller et al., 2000; Sharma et al.,  2009; Wei et al., 2005; Wei et al.,  2008); MS-HRM (unpublished data) |
| ***CDKN2A*** | 2% | 9% | Pyrosequencing (Feng et al., 2007);  MSP (Esteller et al., 2001) |
| ***TP73*** | 2% | 2% | MSP (Esteller et al., 2001); MS-HRM  (unpublished data) |
| ***CDH1*** | 0% | 28% | Pyrosequencing (Feng et al., 2007);  MethyLight (Cho et al., 2010); MSP (Esteller et al., 2001; Prasad et al.,  2008; Toyooka et al., 2002); MS-HRM (unpublished data) |
| ***MGMT*** | 0% | 3% | MSP (Esteller et al., 2001); MS-HRM  (unpublished data) |
