# Supplementary figures and images for: Assessment of DNA methylation profiling and copy number variation as indications of clonal relationship in ipsilateral and contralateral breast cancers to distinguish recurrent breast cancer from a second primary tumour
Source: BMC Cancer. 2015 Oct 9;15:669. doi: 10.1186/s12885-015-1676-0 (PMC4600279; doi:10.1186/s12885-015-1676-0)

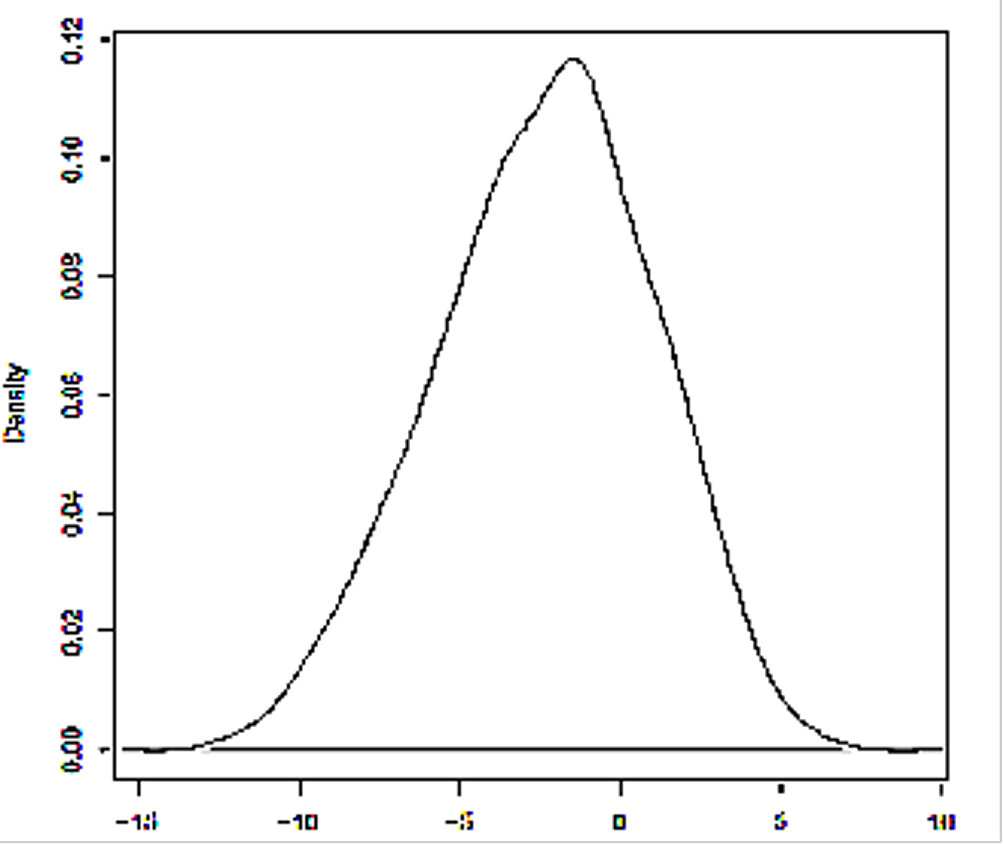

Supplement: Additional file 2: Figure S1. — Normally distributed test statistics generated using empirical null distribution of LRs. N = 3248 and bandwidth = 0.8926. (TIFF 115 kb) [file 12885_2015_1676_MOESM2_ESM.tiff]
